# Improved Contact X-Ray Microradiographic Method to Measure Mineral Density of Hard Dental Tissues

**DOI:** 10.6028/jres.115.006

**Published:** 2010-04-01

**Authors:** B. D. Schmuck, C. M. Carey

**Affiliations:** Polymers Division, National Institute of Standards and Technology, Gaithersburg, MD 20899-8546

**Keywords:** caries, dentin, enamel, erosion, mineral density, mineralization, x-ray microradiography

## Abstract

Contact x-ray microradiography is the current gold standard for measuring mineral densities of partially demineralized tooth specimens. The x-ray sensitive film specified in the last J Res NIST publication on the subject is no longer commercially available.

**Objectives:**

Develop a new microradiographic method by identifying a commercially available film with greater than 3000 lines per millimeter resolution, which is sensitive to x rays, and develop correct film processing for x-ray microradiographic application.

**Methods:**

A holographic film was identified as a potential replacement film. Proper exposure was determined utilizing a thick nickel plate to create test-strips. Film development was bracketed around manufacturer suggestions. Film linearity was determined with aluminum step-wedges. Microradiographs of 100 μm thick tooth sections, before and after acidic challenges, were a final test for film. Magnified images were captured with a digital microscope camera with 0.305 micrometers per pixel resolution.

**Results:**

The appropriate film exposure was 30 min at 80 kV_p_ and 3 mA with a development time of 2 min. Step-wedge experiments show the system to be linear in terms of pixel intensities with respect to x-ray attenuation for normalized pixel intensity values that are 10 % to 90 % offull scale (r^2^ = 0.997) which encompasses the full exposure region of tooth tissue. Enamel sections were analyzed and show distinctive differences between erosion and demineralization. The image capture device resolution of 0.305 micrometers per pixel limits the system resolution.

**Conclusion:**

Use of the identified holographic film when combined with the described processing modifications has resulted in an improved x-ray microradiographic method for the measurement of mineral density of dental hard tissues. The method described can be further improved by using a higher resolution digitization system. The method is appropriate for quantitatively measuring changes in mineral density and erosion. Supported by R01DE14707, NIST, and ADAF.

## 1. Introduction

Contact x-ray microradiography is the primary tool used by researchers to evaluate the mineral density of dental hard tissues [1]. This “gold standard” is particularly important as research regarding changes in mineral density is critical to understanding and preventing (or reversing) human caries: the most prevalent curable disease worldwide [2].

Due to the molecular nature of dental caries, it is critical that caries research be able to measure changes in the mineral density of teeth as early as possible. There are generally four methods utilized for this purpose. Polarized light microscopy of cross sections can observe crystal birefringence changes and thus measure the lesion depth for thin slices. Generally only qualitative information about mineral density, in the form of pore volume, is obtainable with this method [3–7].

Regarding profilometry methods, Attinet al. [8] recently reiterated that “it should be respected that profilometrical determinations do not satisfactorily reflect the mineral loss…” furthermore, profilometry is extremely technique sensitive with results differing between laboratories [8–9]. Another method, surface microhardness measurements, can be correlated to mineral density with the application of certain assumptions, yet it is an indirect measurement and difficult to calibrate against a mineral density microhardness standard. Furthermore, surface microhardness may not be used to investigate natural caries (subsurface lesions) due to its requirement for homogeneous tissue [3]. Recent studies have cast doubt on any correlation between hardness and mineral density in the lower half of the scale [10]. Outside of these limitations microhardness seems to be an acceptable method for early lesions [3, 6–7]. Ultimately, x-ray microradiography is the only current method that determines mineral density directly (via attenuation of x-ray energy by the mineral), and as such it has long been known as the “gold standard” for mineral density measurements [1, 3–7, 10].

However, the x-ray sensitive film previously used for contact microradiography has been discontinued and has been out of production for several years. Existing laboratories have been able to continue work with their own stockpiles but these will eventually be depleted and new laboratories are not able to enter this research arena [11]. Thus, there is a clear need for a new film and improved methods allowing contact microradiography to continue as the gold standard for mineral density measurements of dental hard tissues. This paper identifies a new film and incorporates new developments in digital image analysis.

## 2. Methods

### 2.1 Overview

Tooth tissue is cut into thin parallel slices and imbedded in x-ray transparent epoxy with a geometric positioning marker, leaving a thin tooth edge exposed after polishing. At this point one x-ray microradiograph is taken as a “before” image. Experimental conditions are applied to the tooth samples and x-ray microradiographs are taken during the experiments and at the end as an “after” image. These images are converted from high resolution holographic film into digital images andmined for their mineral density data. Once the raw data are electronically available, “before,” “during,” and “after” microradiographs are compared to show mineral density changes. With proper analysis of mineral density changes, one can distinguish between erosion and demineralization [12–13].

### 2.2 Film

The film used in this study was VRP-M holographic film (Slavich, Lithuania). This film has a grain size of about 0.04 μm, resolving power greater than 3000 lines per millimeter, and is designed for laser holographic imaging (Slavich specification documentation). With grain size and resolving power greatly smaller than the wavelength of visible light (mandatory for the film to function for its designed purpose of holography), the film’s performance should surpass that measurable with an optical system. The discontinued film that was most commonly used in the past was Kodak Professional Film SO343 (Eastman Kodak Co., Rochester, NY).

### 2.3 Tooth Tissue Preparation

**Fig. 1:**
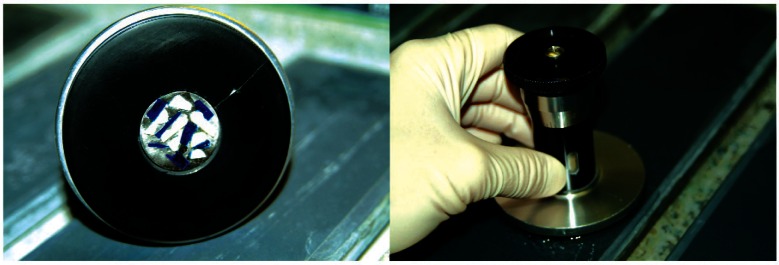
Tooth slices and inked glass cover slips glued to piston portion of microtome (left) and microtome in use on sanding block (right).

Caries-free human teeth were wet cut into slices about 250 μm thick using a diamond blade (Buehler, Lake Bluff, IL) on a Buehler Isomet saw at the highest speed setting. Tooth slices were fixed to the bottom of the piston portion of a sanding microtome (Edmond Scientific, Tonawanda, NY) with Instant Krazy Glue (Elmers, Columbus, OH). Number 1½ size (nominally 170 μm thick) glass microscope cover slips were broken and fixed to the piston between tooth samples and marked on the outward facing side with permanent ink. Samples were wet sanded and polished with 400 and 600 grit silicon carbide abrasive papers (Mager Scientific, Dexter, MI) until ink on the glass cover slips began to wear, indicating a thickness matching that of the cover slip. A small pencil mark was made on a portion of the tooth tissue not intended for use with experimentation. The piston was removed from the microtome and suspended in technical grade acetone until the adhesive was fully dissolved. Tooth samples were washed three times in acetone and then allowed to dry. Tooth samples were then fixed to the microtome piston with the pencil mark down so that final samples would be parallel. The sanding process was repeated with number 0 nominal size glass cover slips so that the final samples were parallel and 100 μm thick (Fig. 1).

### 2.4 Epoxy Mixing and Curing

Epoxy Epon Resin 828 was mixed with Epi-CureCuring Agent 3234 (both Miller-Stephenson, Danbury, CT) according to manufacturer recommendations in a 113:10 ratio by mass. The mixture was stirred 2 min in a 10 mL disposable polystyrene beaker (Fisher Scientific, Pittsburgh, PA) with smooth strokes to avoid creating air bubbles in the epoxy. The epoxy was allowed to sit 1 min allowing a majority of the air bubbles to escape the matrix. This ratio of epoxy and catalyst has a practical fluidity of 15 min and a working time of 30 min. When this epoxy is placed in a 50 ºC oven, it sets within 2 h and effectively cures after 18 h.

### 2.5 Geometric Fixing Mechanism

**Fig. 2:**
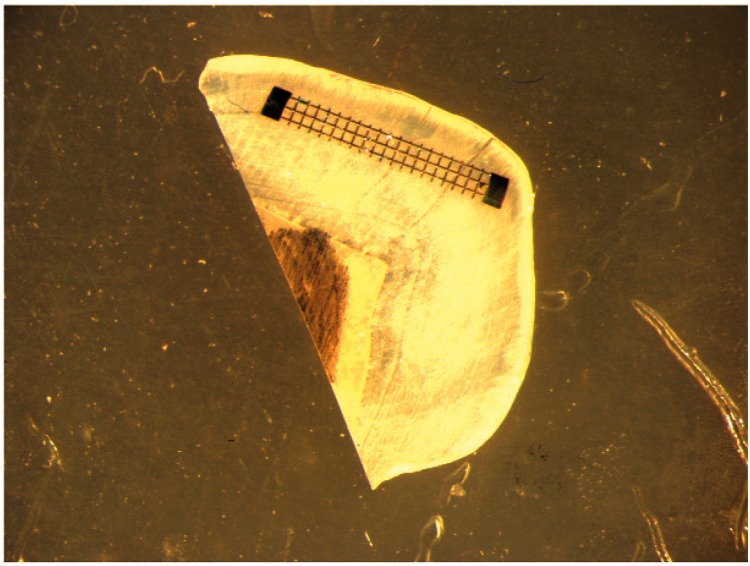
Thinned tooth sclice (pencil mark showing) with cut grid ready for epoxy mounting.

200 mesh Maxtaform Copper/Rhodium transmission electron microscopy (TEM) grids (Electron Microscopy Sciences, Hatfield, PA) were cut under a stereomicroscope into rows encompassing at least one full grid (127 μm pitch, 103 μm hole, and a 24 μm bar —manufacturer numbers reported as ± 0.005 μm). These grid-rows were positioned on tooth sample surfaces parallel to the edge of interest and fixed with a small drop of epoxy. Epoxy was allowed to set before proceeding further (Fig. 2).

### 2.6 Sample Mounting

Epoxy was poured to half fill a rectangular mold and allowed to set until thick. Tooth samples with mounted TEM grids were then placed with enamel and grid parallel; close to the edge of the mold. Freshly mixed epoxy was then poured over tooth samples until the mold was full. The filled mold was placed in a 50 ºC oven until all epoxy had cured.

### 2.7 Final Sample Preparation

**Fig. 3:**
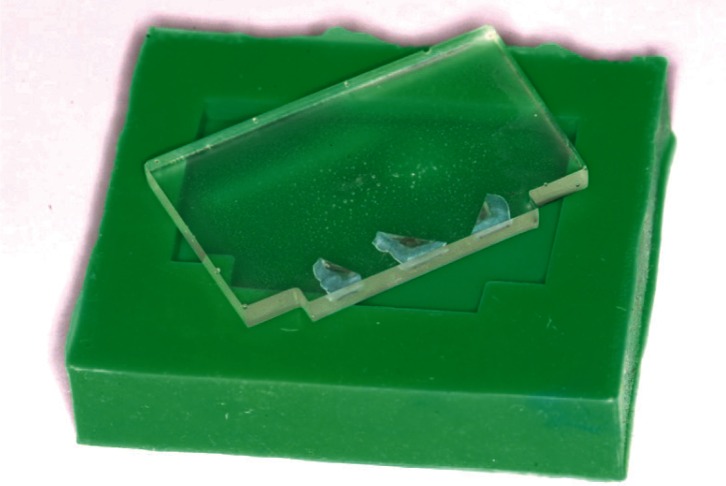
Cured epoxy with embedded tooth samples resting above application specific mold.

Once cured, the epoxy block holding the tooth samples was sanded on a polishing wheel with 600 grit SiC sandpaper until the enamel surface was exposed. Polishing was then performed with 800, 1200, and 2400 grit SiC sandpaper. Care was necessary to keep the sample exactly perpendicular to the wheel so that no bevel could form and cause a false mineral density gradient in the x-ray microradiograph. The exposed enamel edge was rubbed with acetone to be sure that no cured epoxy might have been smeared across the enamel surface during the polishing process (Fig. 3).

## 3. X-Ray Processing

The prepared samples were placed directly on top of the emulsion side of the VRP-M holographic film adjacent to an aluminum step-wedge and enclosed in a light-blocking paper-topped x-ray sample holding tray. The tray was then placed in the center of the first shelf in a Faxitron model #43655A x-ray source cabinet (Hewlett-Packard, Palo Alto, CA), which is about 30 cm from the source, and exposed to Cu radiation for 30 min at 80 kV_p_ and 3 mA. Exposed film was developed for 120 s in JD-2 developer (Integraf, Kirkland, WA), rinsed with D.I. water for 30 s, placed in 1 % mass fraction acetic acid as a stop bath for 60 s and washed for 10 min in flowing tap water. The washed film was then placed in a Form-A-Flo solution (Photographers’ Formulary, Condon, MT) and air dried overnight. Once dry, the film was trimmed to appropriate size and taped flat, emulsion side up and around all four edges, to a clean glass slide and labeled.

## 4. Film Digitization

Film fixed to the glass slide was observed with transmitted light under a Leica MZ16 stereo microscope (Leica Microsystems, Bannockburn, IL). Digital images were captured with an Evolution MP-5.0 digital microscope camera (Media Cybernetics, Silver Spring, MD) with 12 bit grayscale values for each pixel in the 2560 pixel by 1920 pixel image and stored in uncompressed tiff file format. Spatial resolution was determined by quantifying the number of pixels between marked positions on a stage micrometer within the field of view at each optical magnification setting used. Measurement features on the stage micrometer are NIST traceable and accurate to within 0.1 %. For our purposes spatial resolution is reported as micrometers per pixel and is considered to be the aggregate Type B uncertainty for the optical/digital system.

## 5. Image Analysis

Optically magnified digitized x-ray sample images were analyzed with the ImageJ [14–15] software package (U.S. National Institutes of Health, Bethesda, MD). A rectangular selection was made between gridlines and perpendicular to the tooth surface; starting in unaffected enamel, passing through the demineralized zone, and extending outside the tooth surface. The “Plot Profile” option was then used to average pixels in a line and to generate and plot the grayscale profile from the exterior to the interior of the tooth which has a direct correlation to mineral density. These values were then exported to a spreadsheet for further analysis. Detailed instructions regarding these functions are described in the ImageJ documentation and tutorials [14].

## 6. Image Data Comparison

**Fig. 4:**
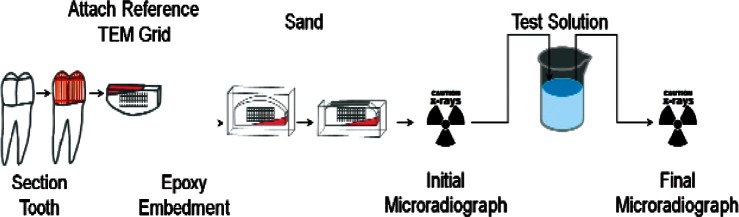
Method overview for dental hard tissue preparation, measurement, and testing.

A key use of the TEM grid epoxied to the surface of the tooth is to provide a positional reference point for comparing the same tooth sample’s x-ray image before, during, and after treatments to the tooth sample. Profiles are collected from before, during, and after in the same grid columns. This data is plotted within the spreadsheet and offset until the grayscale maximum peaks, due to the fixed metal grid, are aligned. The grayscale values are normalized for each data set by setting the black background to 0 % and the unaffected mineral to 100 % mineral density. After normalization, the before and after mineral density profiles are thenplotted on the same graphs. The distance between the before mineral gradient and the intersection of a line of that same slope with the 20 % mineral density point on the after mineral density profile has been defined as erosion. Further mineral density loss that penetrates deeper into the tooth, but has not removed more than 80 % of the mineral density is defined as demineralization. Due to the lack of intuitively meaningful area based parameters [1] it is reasonable to report the lesion depth at a certain percentage of tooth demineralization (Fig. 4).

## 7. Results

### 7.1 Film Linearity

**Fig. 5:**
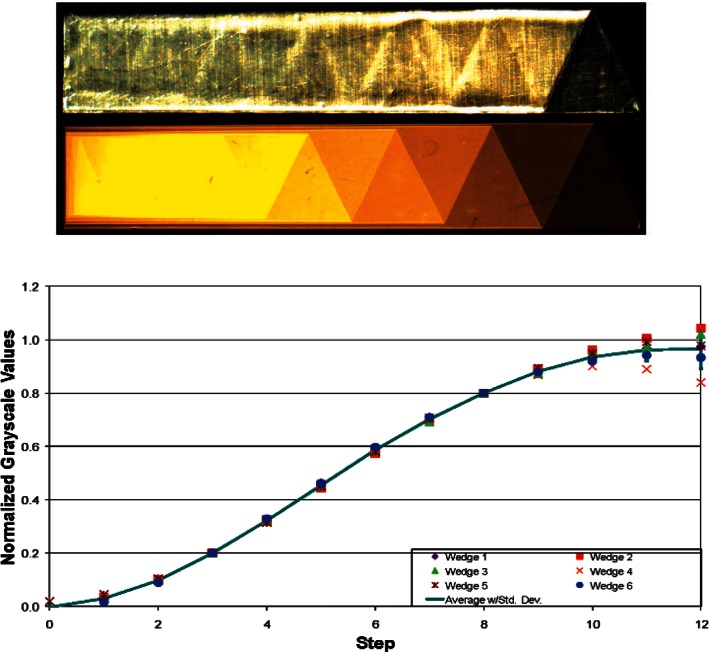
Step wedges. a: Image of a folded aluminum step wedge. b. Radiograph of the same step wedge. c. Normalized Grayscale Values vs. Step-wedge. Composite data from step-wedge experiments with grayscale normalization at 20 % and 80 %. Each data point is an average of 6 separate radiographs of that step of the wedge (error bars smaller than point size). The line is the mean of those normalized values for each separate wedge with standard deviation as error bars. Film is linear between 10 % and 90 % (R^2^ = 0.9959).

Six microradiographs were taken of six step-wedges. The grayscale values recorded from the steps provide information on the linearity and consistency of x-ray attenuation measurements that are representative of the entire process from film exposure to digitization. Slight variations in temperature and freshness of the developing solution, as well as difficulty in obtaining an exactly repeatable light intensity for the digitization process, will give rise to some brightness and contrast differences between separate microradiographs. One may make use of the fact that brightness and contrast are linear modifiers (intercept and slope respectively) of observed grayscale intensity. Therefore, a linear normalization of the grayscale should be able to account for expected differences between microradiographs. However, with an exposure time of 30 min from an energy source (copper K*_a_*x-ray) well outside the spectral range the film was designed for (green light sensitive–500 nm to 550 nm wavelength photons), reciprocity failure is anticipated both in terms of film exposure (a 15 min exposure will be more than half, rather than exactly half of, a 30 min exposure) as well as relative exposure on the film (non-linearity at the lightest and darkest regions of the film) [16]. Due to expected non-linearity at the maximum and minimum grayscale values, the linear normalization points were taken at 20 % and 80 %. Normalization was performed on the 6 separate radiographs containing each of the 6 unique step wedges, and the repeat measurements of each individual step wedge was averaged. The 6 normalized averaged step wedges were then compared to each other and show a linear response for the film between 10 % and 90 % of the total grayscale values with a linear correlation coefficient (r^2^) of 0.9959 (Fig. 5). This compares favorably to the previously reported microradiographic film which, required non-linearregression for grayscale evaluation [1]. A linear calibration has the distinct benefit of allowing an internal relative calibration (at zero and sound tissue mineral densities) which allows for comparison of the sample before and after experimental treatment without the use of a step-wedge (which would be required for standard thicknesses in a non-linear film). The step wedge is included with all samples to assure that the film and processing remains linear in the relevant grayscale range for the experiments. Also, a step wedge is a useful tool for conversion to density units [3].

## 8. Demineralization Measurements

**Fig. 6:**
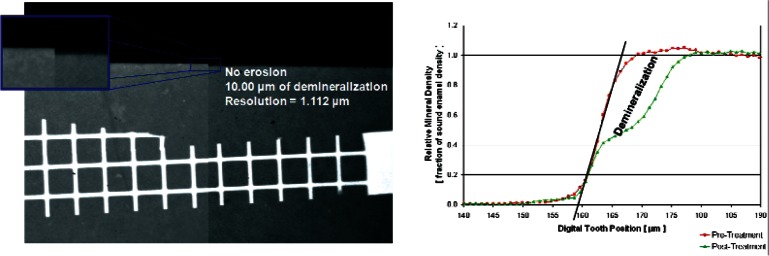
Demineralization without erosion. Radiograph before and after demineralization cycling as aligned images (left) and plotted as relative mineral density versus distance (right). Distance direction is normal to tooth surface.

Enamel sections were exposed to 30 mmol/L phytate as a surface permselectivity modifying pretreatment [17] and were then kept in a continuously flowing solution of 0.25 mL/min artificial saliva [18] containing 57.4 μmol/L sodium fluoride (1.0 ppm fluoride). Enamel sections were exposed to three acid attacks per day which modeled a Stephan curve [19] within the flow chamber. The second microradiograph was taken after a period of two weeks. Both microradiographs were taken with medium optical magnification, a typical compromise between spatial resolution and field of view, yielding a spatial resolution of 1.11 micrometers per pixel. It was found that there was zero erosion, but that demineralization proceeded 10.00 μm into the tooth. Multiple measurements of the same sample were performed, but an unreasonably small number was calculated for standard error (0.0002 μm). This small number for error is an artifact of the discretenature of pixel measurements when the measured component lands on the same pixel with almost every repeat measurement. When standard error is smaller than spatial resolution, it indicates that the system is not able to reach the limits of the film in its current configuration. Higher optical magnification or a better digital capture device would contribute to increased sensitivity. Fig. 6 shows side by side comparison of the two microradiographs and the grayscale analysis. The spatial measurement uncertainty that is attributed to the film’s grain size will not be known until the digitizing process is of a higher resolution than the film itself.

## 9. Erosion Measurements

**Fig. 7:**
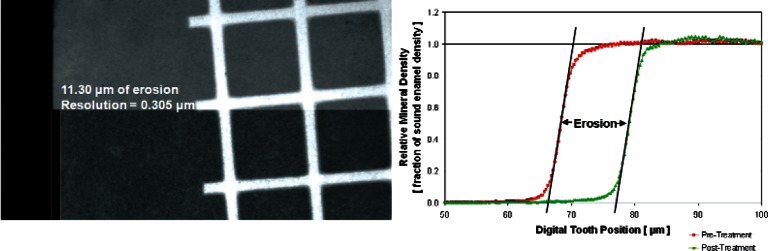
Erosion without demineralization. Radiograph before and after four hour erosion challenge as aligned images (left) and plotted as relative mineral density versus distance (right). Distance direction is normal to tooth surface.

X-ray microradiographs were taken of the prepared enamel sections before and after treatment with an erosive challenge (acidic wash) for 4 h. In this situation there was erosion of 11.3 μm of tooth surface with zero demineralization and the image was digitized at the maximum optical magnification yielding a spatial resolution of 0.305 micrometers per pixel. The system’s maximum optical magnification was clearly an improvement over the spatial resolution used in the demineralization measurement example; however, the standard error was still calculated below the maximum spatial resolution indicating that the error attributed to the manufacturer’s reported resolving power of the film is below our ability to measure. Figure 7 shows the before and after microradiographs and the grayscale profile within the enamel sections.

## 10. Conclusions

This epoxy potting method for contact x-ray micro-radiography using Slavich VRP-M holographic emulsion film digitized under microscope enlargement can be successfully applied to observe and distinguish between erosion and mineralization changes in dental tissue within a linear grayscale system. Repeat step-wedge measurements have shown that the film behaves in a linear fashion in the pertinent exposure regions, although differences in the exposure and development introduce the need to normalize grayscale values before a direct density comparison can be made between two separate images. Standard error values for distance measurements fall below the spatial resolution of the optical-digital system, indicating that the resolution of the digitizing system is the limiting factor. The film itself has enough resolving power that its limits will not be seen until at least the next generation of digitizing system is employed. Overall, VRP-M film was found well-suited to the needs of x-ray microradiography and will likely serve as the film of choice until digital x-ray detectors reach a spectral resolution acceptable for research use. Even then, it may still have considerable utility for such applications as international standards due to greater accessibility.
